# Challenges in the diagnosis and management of atypical fungal keratitis during the COVID-19 pandemic: a case series

**DOI:** 10.1099/acmi.0.000570.v3

**Published:** 2023-08-08

**Authors:** Dideeya Ali, Ajith Vijayan, Kashinatha Shenoy, Ann Tresa Antony, Reshmi Ramachandran

**Affiliations:** ^1^​ Department of Ophthalmology, Pushpagiri Institute of Medical Sciences and Research Centre, Thiruvalla, India; ^2^​ Department of Microbiology, Pushpagiri Institute of Medical Sciences and Research Centre, Thiruvalla, India

**Keywords:** Atypical fungal keratitis, COVID-19, *Scedosporium*, *Aureobasidium*, *Trichosporon*, *Purpureocillium*

## Abstract

Fungal keratitis is a time-sensitive ocular infection that often requires a high index of suspicion followed by intensive medical/surgical interventions to achieve a successful clinical outcome. COVID-19 pandemic-related restrictions, necessitated the modification of conventional protocols and guidelines associated with the treatment of keratomycosis. We report four cases of atypical fungal keratitis with poorly differentiated clinical characteristics. The challenges faced during their management were (1) the dilemma of clinically differentiating fungal (*Scedosporium and Purpureocillium lilacinum*) and bacterial keratitis; (2) treatment of *Scedosporium* and *Trichosporon* keratitis with natamycin monotherapy; (3) mixed infection of *Candida albicans* and *Aureobasidium pullulans* and continuing medications before rescraping the corneal ulcer against the recommended treatment guidelines; (4) phenotypic identification and differentiation among morphologically resembling fungi; and (5) decision making arising out of disparities between KOH and fungal culture results. Three patients responded well to conservative treatments. The fourth patient underwent therapeutic keratoplasty but was lost to follow-up due to travel**-**related pandemic restrictions. This case series seeks to broaden the clinician’s knowledge of rare and emerging moulds as presumptive aetiologies of keratomycosis. It also intends to emphasize the significance of early microbiological investigations, (direct microscopy and culture), in resource-limited settings, for initiating empirical treatment for a better visual prognosis.

## Data Summary

No data was generated or reused.

## Introduction

Keratomycosis or fungal keratitis (FK) is an invasive infection of the corneal stroma. Corneal opacification secondary to keratitis, is also known to be the second most common cause of blindness after cataract [1]. Developing, tropical and subtropical countries have reported almost half of the world’s FK [2]. In India, corneal ulcers are caused by bacteria and fungi in equal proportions. Cyril *et al*. [3] inferred that only 66 % of corneal specialists were able to clinically distinguish between fungal and bacterial corneal ulcers. Hence, the rationale for empirical therapy must be reviewed. Early diagnosis and treatment of FK also avoids intraocular involvement and complications [4–6]. This underlines the importance of diagnostic mycological investigations during the initial examination of the patient. Contemporary investigators have reported *Aspergillus* spp. and *Fusarium* spp. as the most common etiological agents for fungal keratitis [7–10]. Rarer fungal pathogens like *Acremonium* spp.*, Alternaria* spp.*, Penicillium* spp*., Bipolaris* spp.*, Curvularia* spp*., Phialophora* spp.*, Blastomyces* spp*., Sporothrix* spp*., Exophiala* spp., *Scedosporium* spp., *Cylindrocarpon* spp*., Purpureocillium* spp., *Lasiodiplodia theobromae, Metarhizium anisopliae* and *Pythium insidiosum* have also been described [11]. Conventional treatment protocols had been bypassed during the COVID-19 pandemic owing to patient’s financial and travel constraints and the hospital’s logistic constraints for prioritizing in-patient admissions, based on their COVID-19 status. Accordingly, patients preferred to access the nearby healthcare facilities post-lockdown. This provides an opportunity to expand the knowledge of the healthcare providers to the rarer aetiologies of common clinical conditions. In this case series, we share our unusual experiences of diagnosing and treating four atypical cases of fungal keratitis during the COVID-19 pandemic.

## Case presentation

### Case 1

A 68-year-old man with type 2 diabetes mellitus (T2DM) and coronary artery disease (CAD) on treatment, presented to our outpatient department (OPD) with complaints of swelling of the left eye and difficulty in opening the eyes for 3 days. On examination, his best corrected visual acuity (BCVA) in the right eye was 6/18 and left eye counting fingers ½ m. The right eye was normal following a slit lamp examination. There was a central circular ring-shaped 3×3 mm anterior stromal infiltrate around a pre-existing central scar in the left eye. Patient was administered 0.5 % topical Moxifloxacin and 1 % Amikacin every 2 h, along with 2 % Homatropine twice daily after a provisional diagnosis of bacterial keratitis. However, potassium hydroxide (KOH) mount of the corneal scraping revealed septate hyphae. Hence, the patient was treated with 5 % Natamycin every 2 h. The ulcer healed completely with scarring on day 30. *Scedosporium* spp. was isolated from the fungal culture (Table 1). BCVA improved to counting fingers (CF) 2 m partly due to central scarring and diabetic retinopathy.

**Table 1.** Diagnostic mycology findings of the cases of atypical fungal keratitis [ 20]

Clinical cases	Direct microscopy findings – KOH mount	Colony morphology of the isolate on SDA at 37 °C/28 °C	Smear findings from isolated colonies on SDA (Lactophenol Cotton Blue tease mount preparation/Gram stain)	Final Identification of the isolate
1	Septate hyphae	Greyish-white cottony colonies on the obverse with reverse black pigmentation (Fig. 3a)	Hyaline septate hyphae with percurrent, lateral and terminal conidiogenous cells [21]. *Graphium* conidiation (septate hyphae with long conidiophores cemented to form synnemata) (Fig. 3b)	*Scedosporium species complex*
2	Septate hyphae	Dry cream-coloured colonies with a crumb-like appearance adhered to the agar (Fig. 4a)	Abundant arthroconidia with blastoconidia, true hyphae and pseudo hyphae (Fig. 4b)	*Trichosporon asahii*
3 (Isolate 1)	Sparsely septate hyphae	Obverse brownish black moist to creamy colonies with reverse brown to black pigmentation (Fig. 5a)	Darkly pigmented arthroconidia seen. Scanty hyaline, smooth walled ellipsoidal conidia along the hyphae (Fig. 5b)	*Aureobasidium pullulans*
3 (Isolate 2)	Yeast-like cells with hyphae	White coloured creamy to pasty colonies at 37 °C (Fig. 5c)	Gram positive oval budding yeast cells with hyphae and pseudo hyphae (Fig. 5a)	*Candida albicans*
4	No fungal elements	Obverse powdery, cottony, velvety pink to lilac-coloured colonies with reverse off-white pigmentation (Fig. 6a)	Septate hyaline hyphae. Branched conidiophores bearing metulae with tapering phialides bending away from the axis of the conidiophore. Oval to fusiform catenulate phialoconidia (Fig. 6a)	*Purpureocillium lilacinum*

### Case 2

**Fig. 1.**
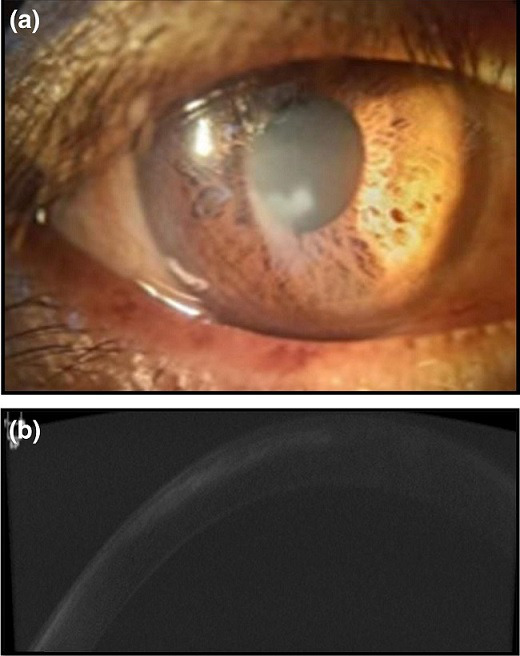
(a) Healed thin corneal scar near the pupillary area (case 2). (b) Anterior segment-optical coherence tomography (AS OCT) of case 2 showing small thin scar in the superficial corneal stroma.

A 48-year-old man with T2DM, presented with a post-traumatic pupil associated with a third nerve palsy involving the right eye. Limitation of elevation of the right eye with the right upper eye lid was noted. Corneal sensation was intact without any other cranial nerve involvement. After 3 weeks of follow up, he developed pain and redness in the same eye. His BCVA was CF 3 m, and cornea showed a 2.5×1.5 mm linear anterior stromal infiltrate with an overlying epithelial defect. KOH mount done on the corneal scraping revealed fungal elements. Therefore, he was treated with topical 5 % Natamycin for every 2 h, 0.5 % Moxifloxacin thrice daily and 1 % Cyclopentolate thrice daily. Ulcer healed completely by day 28. *Trichosporon asahii* was reported from fungal culture (Table 1). BCVA improved to 6/18. (Fig. 1a, b)

### Case 3

**Fig. 2.**
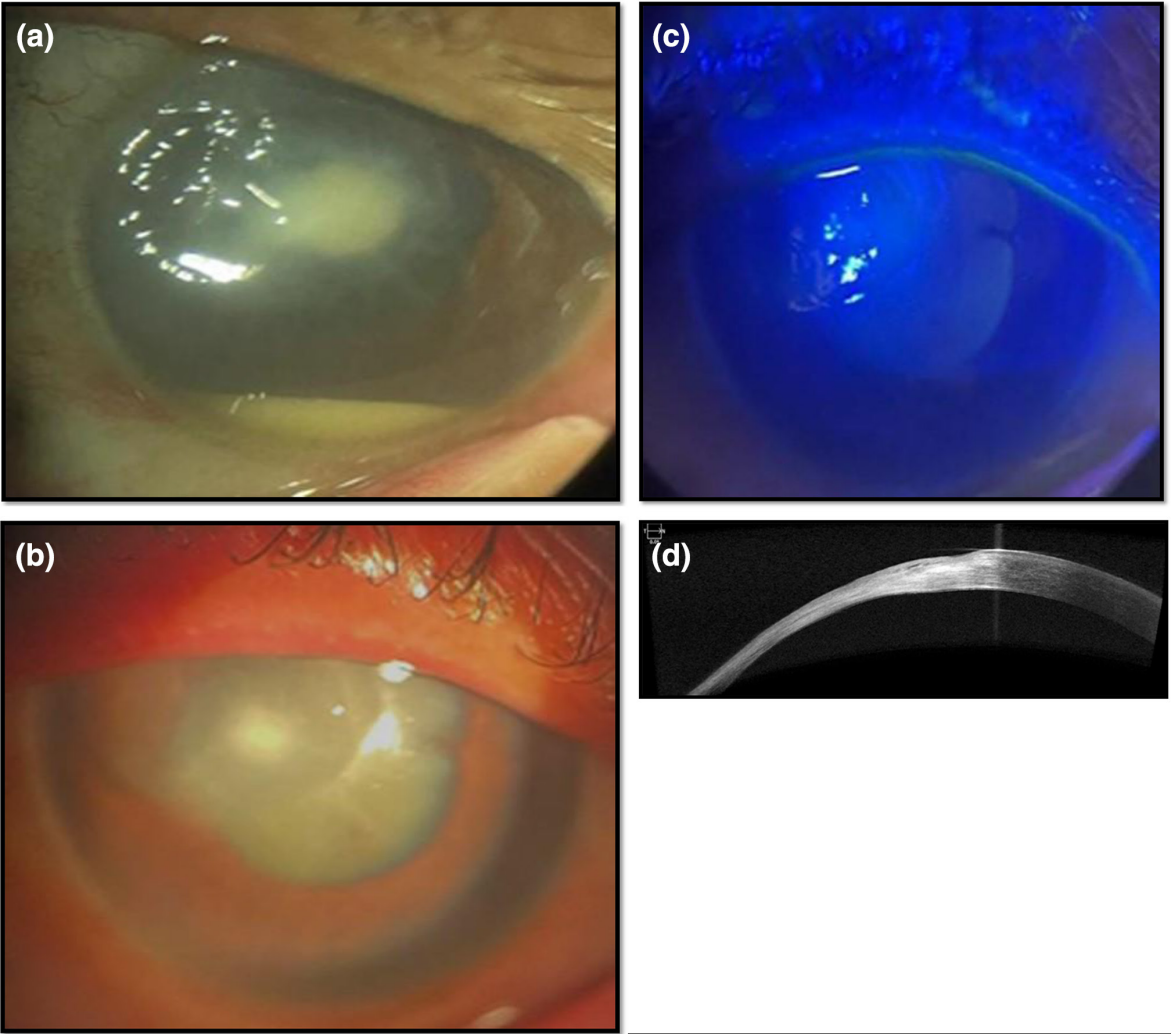
(a) Deep corneal stroma infiltrate and hypopyon (case 3). (b) Healed FK with thinning and leucomatous opacity (case 3). (c) Healed FK, fluorescein staining under cobalt blue filter (case 3). (d) AS OCT showing corneal thinning deep stromal scarring (case 3).

A 76-year-old woman with a history of plant trauma, presented with pain, redness and watering of right eye for 3 days. She was previously being treated for dyslipidaemia and systemic hypertension. Her BCVA in right eye was 6/36 and that in her left eye was 6/12. Cornea of the right eye showed a 4×4 mm paracentral mid stromal infiltrate with surrounding corneal oedema. Empirical treatment was initiated with topical 5 % Natamycin every 2 h,0.3 % Moxifloxacin thrice daily and 2 % Homatropine drops twice daily. On day 20, she developed a 2.5 mm hypopyon after a brief symptomatic improvement (Fig. 2a). KOH mount from the corneal scraping showed sparsely septate hyphae with yeast-like cells. On day 25, treatment was changed to fortified 0.15 % Amphotericin B hourly along with Natamycin. On day 30, the ulcer healed but the hypopyon persisted. Oral Itraconazole 200 mg bd was added to her treatment regimen. As contaminant fungi were isolated from fungal culture, rescraping was done for a second culture on day 35, which grew *Aureobasidium pullulans* and *Candida albicans* (Table 1). The ulcer healed completely on day 60, with paracentral thinning and a leucomatous opacity (Fig. 2b, c, d). Her topical medications were tapered gradually.

### Case 4

A 46-year-old man migrant farmer presented with a history of on-field foreign body injury with pain and redness in the right eye. This was treated with 5 % topical Natamycin and oral Fluconazole 150 mg at another hospital. Unsatisfied with his recovery, he consulted our OPD with an 8×7 mm large non-healing corneal ulcer and a 3.5 mm hypopyon. His BCVA was counting fingers close to face (CFCF) in the right eye and 6/9 in the left eye. Due to the intraocular spread and poor response to parenteral and topical antibiotics and antifungals, he underwent therapeutic keratoplasty. KOH mount done from the patient’s corneal button was negative for fungal elements. Hence, the patient was empirically started on fortified Ceftazidime every 2 h, 5 % Natamycin six times a day, Tobramycin eye drops every 2 h and oral Ciprofloxacin 500 mg. He was advised to review after 3 days. However, we lost our patient to follow up as he returned to his hometown post-lockdown. So, his treatment response also could not be assessed. *Purpureocillium lilacinum* was isolated from the fungal culture (Table 1).

### Diagnostic mycology findings

Corneal epithelium was scraped off from the ulcer, with aseptic precautions, using a sterile no. 11 blade, under direct vision through slit lamp, after instilling 0.5 % Proparacaine anaesthetic eye drops. Direct microscopy of the patient samples was done with 10 % KOH. Corneal scrapings and the resected corneal button (case 4), were streaked in a superficial ‘C’ pattern, on Saboraud Dextrose Agar (SDA), without Chloramphenicol or Cycloheximide, to recover those saprobic fungi, which are not only inhibited by these antimicrobials, but are also implicated in opportunistic infections. The culture plates were incubated, at 37 and 28 °C for 4 weeks, to enable the isolation of dimorphic fungi. Cultures were reviewed every day for the first 2 weeks, and every alternate day, during the third and fourth weeks. Fungal growth observed on the “C streaks'' on SDA, with or without a positive KOH mount from clinical samples, were considered significant. Growth outside the “C streaks'' were regarded as contaminants [9]. Bacterial growth was ruled out by negative cultures done concurrently on HI Media Sheep Blood Agar. Moulds growing on SDA, were identified phenotypically, as per standard laboratory protocols – colony morphology, growth rate, Lactophenol Cotton Blue (LPCB) staining and Riedles Slide Culture Method. Gram stain was done for the identification of isolated yeast colonies on SDA. HI Media CHROMAgar Medium was utilized for the speciation of *Candida* spp. Phenotypic methods – germ tube test, urease and assimilation tests – were also performed for identification of the yeasts. The findings are summarized in Table 1

## Discussion

The World Health Organization (WHO) has included infectious keratitis as one of the many neglected tropical diseases [12]. Among the several risk factors reviewed by Shahram *et al*. [13], corneal trauma accounted for 40–60 % of cases of FK. Notable among others are long-term steroid use, diabetes, use of contact lens, farming, past penetrating keratoplasty and exposure keratitis. In this case series, T2DM (cases 1 and 2), trauma and foreign body exposure during farming (cases 3 and 4) were identified as co-existent and independent risk factors of microbial keratitis. Moreover, alterations in the tear components, commensals and the enzyme activities in a long-term hyperglycaemic ocular microenvironment facilitate fungal adherence, proliferation and deep-layer penetration [14]. Xin *et al*. [15] observed that delayed medical attention-seeking by farmers, especially during harvest season, resulted in a poorer response to antifungal drugs and higher rates of surgery.

### Therapeutic and diagnostic challenges in fungal keratitis

Good communication between the ophthalmologist and the mycologist ensured the isolation of all the aetiological fungi from culture. After initial slit-lamp examination, corneal scraping is done for 10 % KOH mount and fungal culture. Other samples include corneal biopsies (if clinical suspicion persisted despite negative KOH and culture results) and anterior chamber aspirates (if there was intraocular involvement) [13].

The diagnostic utility of a KOH mount had been calculated by Bharati *et al*. [16] on 3298 eye samples with resultant sensitivity, specificity positive and negative predictive values of 99.3, 99.1, 98.5 and 99.6 %, respectively. In the same study, insufficient samples, inexperienced observers and small size of the corneal ulcer yielded negative KOH results.

Fungal cultures remain the gold standard test, especially in resource-limited laboratories. However, the samples have to be incubated for up to 4 weeks. It can also turn out to be false positive (due to the isolation of environmental contaminants) or false negative (due to scanty specimens, poor sampling, previous medications and deep corneal involvement) [13].

In our routine clinical practice, the overlying epithelium along with necrotic material and mucus are debrided, after corneal scraping, for better drug penetration. The antifungal agents are also started hourly for 48 h till the signs of healing are visible. *Candida* infections are treated with 0.15 % Amphotericin B or 1 % Econazole; alternatives are 5 % Natamycin, 2 % Fluconazole and 1 % Voriconazole. Filamentous fungi are treated with 5 % Natamycin or 1 % Econazole or alternatively 0.15 % Amphotericin B 1 % Miconazole or 1 % Voriconazole. Broad spectrum antibiotics prevent bacterial co-infection. Mydriatics or cycloplegics (1 % Cyclopentolate, 2 % Homatropine or 1 % Atropine) prevent posterior synechiae. If there is no treatment response despite patient compliance to the antifungal regimen, there is a possibility of the drug not acting against the implicated fungus. In such situations, the treatment is temporarily suspended for the next 24 h and a repeat corneal scraping is sent for culture. This ensures the recovery of resistant variants of the same or a new fungus and helps the ophthalmologist to suitably modify the treatment regimen [17].

The examination findings in advanced fungal keratitis are indistinguishable from that of bacterial keratitis [7]. Drug-related concerns include limited spectrum of action of available antifungals, poor ocular drug penetration of systemic antifungals, and limited commercial availability of topical antifungals. Drug toxicity and associated ocular complications occur due to the prolonged clinical course causing delayed corneal healing. The fungistatic action of the current antifungals are likely to cause recurrence of FK [3]. Patients' refractory to medical therapy, undergo an expensive keratoplasty with a high risk of transplant rejection, and a donor tissue is hardly ever available [1].

### Case 1

**Fig. 3.**
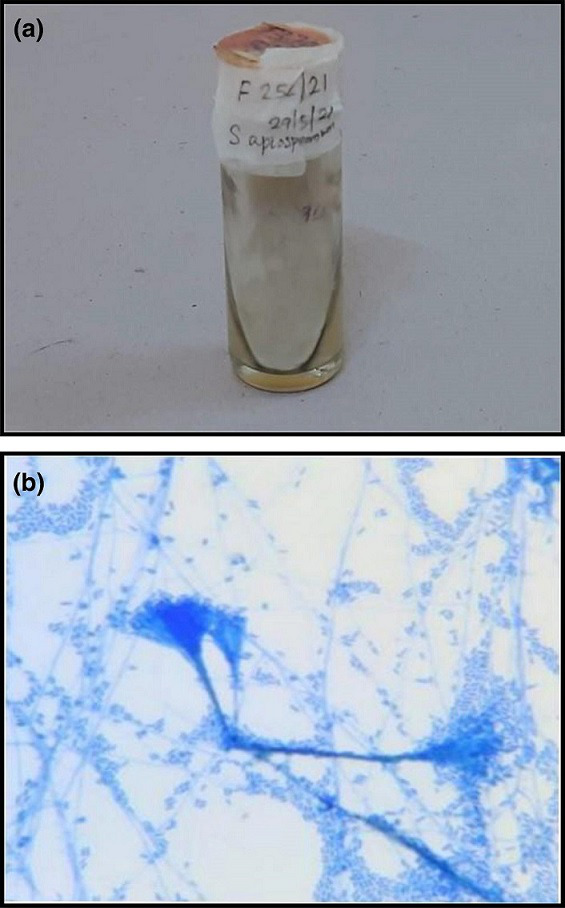
**a,b** Colony morphology and LPCB tease mount preparation (magnification 100x) of *Scedosporium species complex*.

The fungus could have possibly entered as a result of trivial trauma caused by rubbing of the eye and diabetic corneal erosion [18, 19]. The four clinically encountered species belonging to the *Scedosporium species complex* are *S. apiospermum*, *S. boydii*, *S. auranticum* and *S. deboogi* [20]. As all the above-mentioned species appear morphologically similar in culture, we could only identify the synanamorph, *Graphium*, of the fungus along with the conidiogenous cells. (Fig. 3a, b). The latest ECMM-ISHAM-ASM guidelines recommend Voriconazole as the first-line therapy across all patterns of organ involvement [21]. Mycotic ulcer treatment trial ll demonstrated no therapeutic benefit of adding oral Voriconazole to topical antifungal therapy [22]. However, two cases of *Scedosporium* keratitis were treated with topical and oral Voriconazole [18, 23]. Ramakrishnan *et al*. [24] treated *S. keratitis* among seven out of the ten patients with a combination of Natamycin and Fluconazole. Natamycin monotherapy was employed by Rathi *et al*. [25] in five out of eight cases. Our patient also recovered with topical 5 % Natamycin monotherapy alone. Thus, it can be inferred that the treatment must be tailored according to clinical response.

### Case 2

**Fig. 4.**
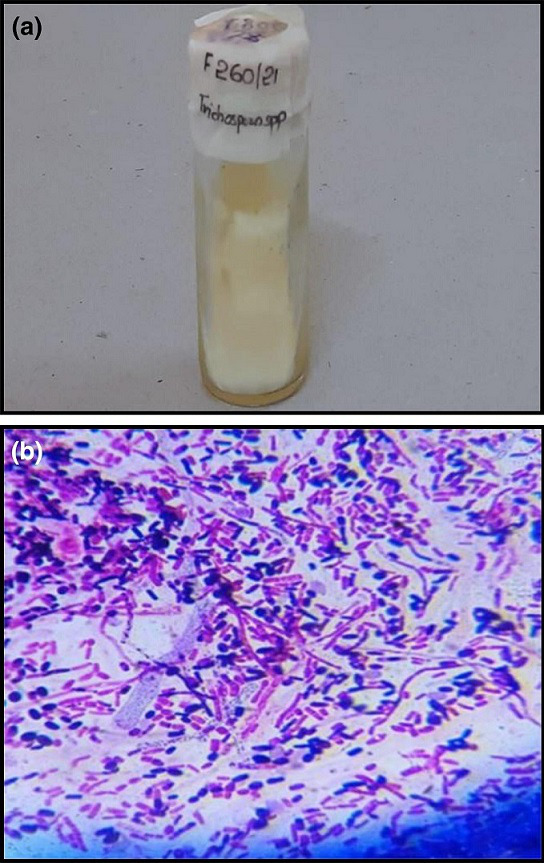
**a,b** Colony morphology and Gram stain (magnification 100x) of *Trichosporon asahii*.

The patient with a history of trauma had no epithelial defects during the preliminary examination. But, with an associated elevation deficit of the right eye with the involvement of the right eyelid, exposure of the cornea during sleep could have predisposed to FK after 3 weeks. *Geotrichum* spp.*,* morphologically resembles *Trichosporon* spp. due to the predominant arthroconidia and hence, it was excluded by urease and assimilation tests. *Trichosporon* spp. (Fig. 4a, b) are known to rarely cause FK in Indian case reports [26, 27]. They are intrinsically resistant to Echinocandins with low MICs to both Voriconazole and Posaconazole. This patient also responded well to empirical 5 % Natamycin monotherapy due to the early initiation of treatment. His ulcer also resolved completely with minimal scarring (Fig. 1a, b).

### Case 3

**Fig. 5.**
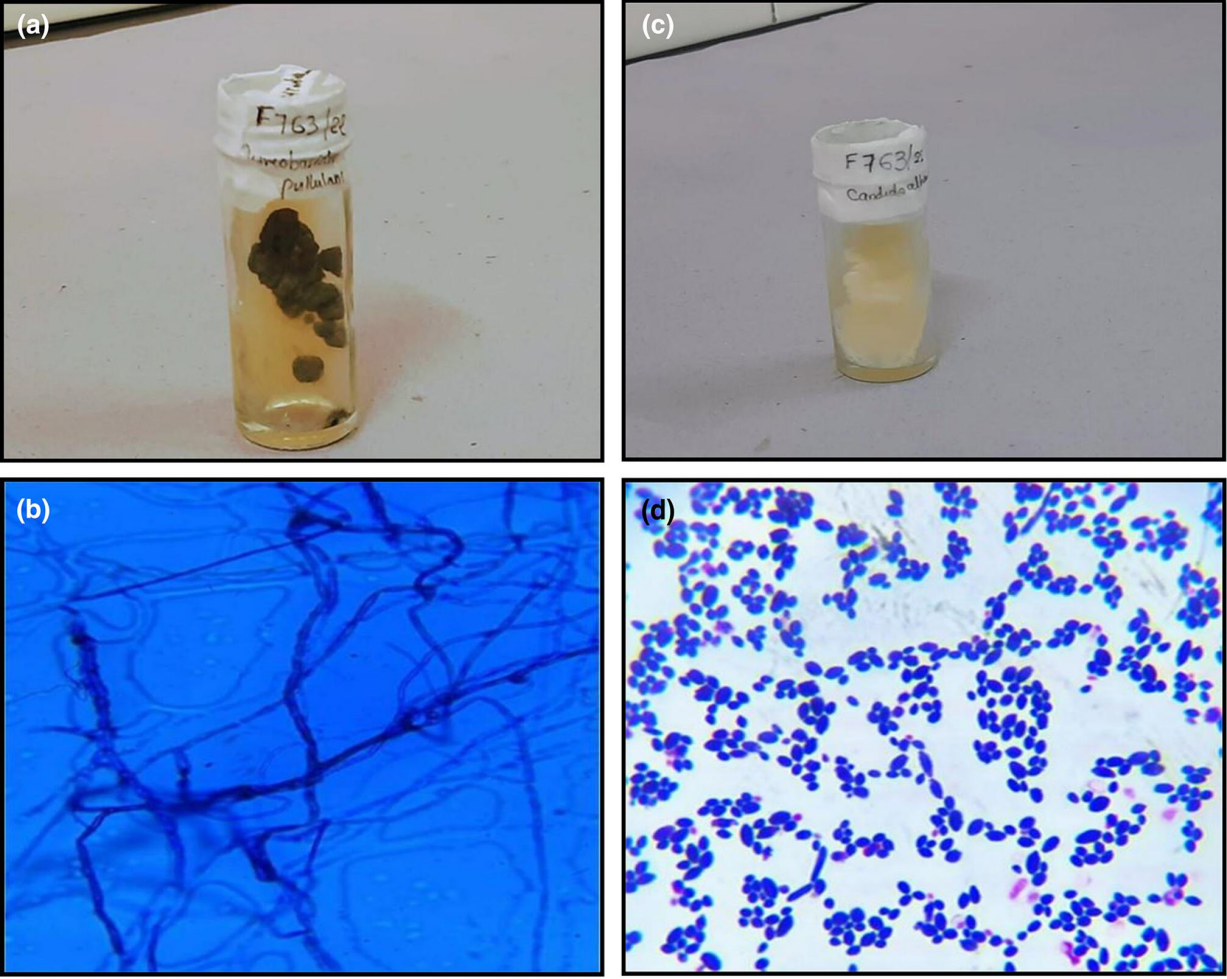
**a,b** Colony morphology and LPCB tease mount preparation (magnification 100 x) of *Aureobasidium pullulans* **c, d** Colony morphology and Gram stain (magnification 100 x) of *Candida albicans*.

Clinical findings were consistent with FK in this dual infection with *Aureobasidium pullulans* and *C. albicans. Candida* keratitis have been reported more in the west than in Asia [28]. Few published case reports also highlight topical steroid use, previous ocular surface diseases or keratorefractive surgeries as risk factors for *Candida* keratitis [29–31]. As this patient had none of these factors, we presume that *C. albicans* may have been acquired from his own flora [32]. The clinical findings of the patient must be corroborated to determine if *Aureobasidium pullulans,* a phaeohyphomycete, is a contaminant in clinical samples or a pathogen. The thin and thick-walled septate dematiaceous hyphae of *A. pullulans* (Fig. 5a, b) can resemble another non-pathogenic phaeohyphomycete, *Hormonema dematioides* [20]. Augustin *et al*. [33] noted good recovery with Fluconazole, Natamycin and repeated scraping. Empirical treatment with topical 5 % Natamycin was started in this case as the patient refused consent for corneal scraping. She consented to the procedure only when her recovery was unsatisfactory. Since contaminants were isolated from the initial fungal culture and hypopyon persisted, rescraping had to be done. Her travel constraints made us deviate from the conventional practice of stopping her medication before rescraping. Itraconazole and 0.15 % fortified Amphotericin B were added. Studies report higher *in vitro* antifungal resistance to Fluconazole, Voriconazole and Echinocandins for *A. pullulans* than Itraconazole, Posaconazole and Amphotericin B [34, 35]. Intravenous preparation of Amphotericin B was reconstituted for topical administration in this patient due to its commercial non-availability. The short shelf life (5–7 days) of the formulations increased the treatment cost and OPD visits of our patient. Even though the patient was non-compliant to 5 % Natamycin, she improved with Amphotericin B and Itraconazole. Nevertheless, delayed treatment and healing caused a dense and larger scar (Fig. 2a-d).

### Case 4

**Fig. 6.**
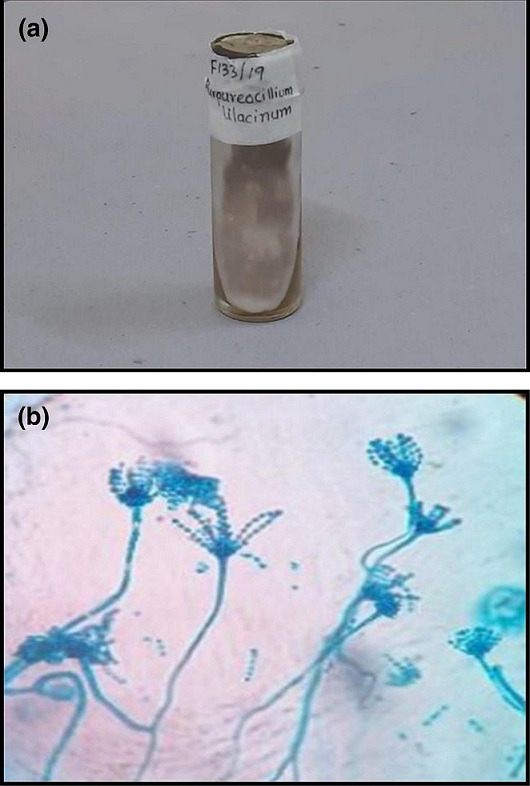
**a,b** Colony morphology and LPCB tease mount preparation (magnification 100x) of *Purpureocillium lilacinum*.

This patient presented with an advanced ulcer due to *Purpureocillium lilacinum* (previously *Paecilomyces lilacinus*) [36]. The slender tapering whorls of phialides of *Purpureocillium lilacinum* were differentiated from the ‘Penicillus'' or ‘brush-like’ phialoconidia of *Penicillium* spp. [20] (Fig. 6a, b). The fungi invades the injured or diseased ocular surface [37] and elicits an innate immune response due to the production of hydrolytic enzymes and the release of mycotoxic peptides such as leucinostatins [38]. Corneal inflammation alters its transparency due to the associated oedema, and changes in the refractive index affect vision [39]. Several case series [40–42] mention that the ocular tropism of this fungi occurs due to tropical climate, contact lens use and exposure to vegetable matter. Though the probability of FK was higher, it was still clinically challenging to rule out bacterial keratitis. The fungus is resistant to Amphotericin B, Fluconazole and Echinocandins and sensitive to second-generation triazoles [37, 43, 44]. KOH was negative (possibly due to the absence of viable fungi in the scanty sample) and no response to empirical antifungal therapy was noted. Medical line of treatment has been successful only in a few cases [43–46]. Foreseeing the possibility of intraocular spread, the patient underwent penetrating keratoplasty. But, the outcome of postoperative management could not be assessed as the patient could not be followed up.

In this case series, the central ulcers (noted in cases 1 and 2) and hypopyon (noted in case 3) could be successfully managed without admitting the patients, despite COVID-19-related constraints. Butt *et al*. [47] also concur that there were no significant differences in the treatment outcomes of admitted and self-medicating microbial keratitis patients.

There were a few limitations in this case series. The use of confocal microscopy could have avoided repeated scraping of the cornea and decreased the patient visits in case 3. We were unable to follow up on case 4 due to lockdown-related constraints. Due to the ongoing standardization of testing protocols in our laboratory, antifungal susceptibility testing, that could have influenced the treatment outcomes of all our patients was not done. It is in this context that interactions with the diagnostician can help prioritize culture over KOH testing in resource-limited settings for scanty samples, depending on the stage of presentation of the corneal ulcer. Fungi may also be directly identified from scanty samples by molecular methods like Conventional PCR followed by post-amplification procedures [restriction enzyme digestion and analysis, single-base extension, hybridization probes or capillary-based molecular sequencing, and single-strand conformational polymorphism (SSCP)] [13]. Molecular testing is not always feasible due to logistic constraints in developing countries. Therefore, the importance of phenotypic identification up to the genus level, which was done for all our patients in this case series, cannot be overlooked.

Tawde *et al*. [48] have tempero spatially correlated the source of fungi causing keratitis by environmental sampling. The state of Kerala receives an annual rainfall of 300 cm and provides a moist environment for the thriving fungi [49]. Furthermore, the devastating floods in 2018 and 2019, as well as the excessive rainfall in the following years, have expanded the ecological niche of the fungi to newer areas. This could have possibly triggered regional spikes in the occurrence of atypical keratomycosis. So, we believe that conventional treatment guidelines need to be individualized to the stage of the presentation of fungal keratitis. In this regard, a trend analysis tracing the atypical fungi causing keratomycosis to their environmental habitats, may be undertaken to guide further modifications in the treatment protocols.

## Conclusion

Early diagnosis and treatment, backed by good communication with the microbiologist, more so during contingencies, help in prevention of the ocular complications and improvement of the visual outcome. Disparities between the findings of KOH smears and fungal cultures must be discussed with the clinician so that the plan of care may be revised according to the patient’s clinical profiles. Hence, microbiological cultures are mandatory to identify the etiological agents of microbial keratitis because the regional climatic changes may dictate the ecological niche of the microbes.

## References

[1] Chander J (2012). Miscellaneous Mycoses, Related Topics.

[2] Ahmadikia K, Aghaei Gharehbolagh S, Fallah B, Naeimi Eshkaleti M, Malekifar P (2021). Distribution, prevalence, and causative agents of fungal keratitis: a systematic review and meta-analysis (1990 to 2020). Front Cell Infect Microbiol.

[3] Dalmon C, Porco TC, Lietman TM, Prajna NV, Prajna L (2012). The clinical differentiation of bacterial and fungal keratitis: a photographic survey. Invest Ophthalmol Vis Sci.

[4] Farias R, Pinho L, Santos R (2017). Epidemiological profile of infectious keratitis. Rev Bras Oftalmol.

[5] Lakhundi S, Siddiqui R, Khan NA (2017). Pathogenesis of microbial keratitis. Microb Pathog.

[6] Vajpayee RB, Gupta SK, Bareja U, Kishore K (1990). Ocular atopy and mycotic keratitis. Ann Ophthalmol.

[7] Prajna VN, Prajna L, Muthiah Srinivasan (2017). Fungal keratitis: the Aravind experience. Indian J Ophthalmol.

[8] Manikandan M (2019). Epidemiology, rapid detection, and antifungal Susceptibilities of *Fusarium* and *Aspergillus* isolates from corneal Scrapings volume 2019, article ID. Biomed Res Int.

[9] Tilak R, Singh A, Maurya OPS, Chandra A, Tilak V (2010). Mycotic keratitis in India: a five-year retrospective study. J Infect Dev Ctries.

[10] Satpathy G, Ahmed NH, Nayak N, Tandon R, Sharma N (2019). Spectrum of mycotic keratitis in north India: Sixteen years study from a tertiary care ophthalmic centre. Journal of Infection and Public Health.

[11] Suman S, Kumar A, Saxena I, Kumar M, Rodriguez-Garcia A, Hernandez-Camarena JC (2021). Infectious Eye Diseases - Recent Advances in Diagnosis and Treatment.

[12] Ung L, Acharya NR, Agarwal T, Alfonso EC, Bagga B (2019). Infectious corneal ulceration: a proposal for neglected tropical disease status. Bull World Health Organ.

[13] Mahmoudi S, Masoomi A, Ahmadikia K, Tabatabaei SA, Soleimani M (2018). Fungal keratitis: an overview of clinical and laboratory aspects. Mycoses.

[14] Dan J, Zhou Q, Zhai H, Cheng J, Wan L (2018). Clinical analysis of fungal keratitis in patients with and without diabetes. PLoS One.

[15] Jin X, Feng J, Sun N, Jin H, Wang J (2022). A 5-year retrospective analysis of the risk factors, treatment, and prognosis of patients with fungal keratitis in Heilongjiang, China. Am J Ophthalmol.

[16] Bharathi MJ, Ramakrishnan R, Meenakshi R, Mittal S, Shivakumar C (2006). Microbiological diagnosis of infective keratitis: comparative evaluation of direct microscopy and culture results. Br J Ophthalmol.

[17] Bowling B (2015). Kanski’s Clinical Ophthalmology. 8th ed.

[18] Nulens E, Eggink C, Rijs AJMM, Wesseling P, Verweij PE (2003). Keratitis caused by *Scedosporium apiospermum* successfully treated with a cornea transplant and voriconazole. J Clin Microbiol.

[19] Fadzillah M-T, Ishak S-R, Ibrahim M (2013). Refractory *Scedosporium apiospermum* keratitis successfully treated with combination of amphotericin B and voriconazole. Case Rep Ophthalmol Med.

[20] Walsh TJ, Hayden RT, Larone DH (2018). Larone’s Medically Important Fungi- A Guide to Identification.

[21] Hoenigl M, Salmanton-García J, Walsh TJ, Nucci M, Neoh CF (2021). Global guideline for the diagnosis and management of rare mould infections: an initiative of the European Confederation of Medical Mycology in cooperation with the International Society for Human and Animal Mycology and the American Society for Microbiology. Lancet Infect Dis.

[22] Prajna NV, Krishnan T, Rajaraman R, Patel S, Srinivasan M (2016). Effect of oral voriconazole on fungal keratitis in the mycotic ulcer treatment trial II (MUTT II): a randomized clinical trial. JAMA Ophthalmol.

[23] Yildirim N, Oz Y, Aydin Yaz Y (2022). Scedosporium apiospermum keratitis treated with voriconazole. Eur Eye Res.

[24] Ramakrishnan S, Mandlik K, Sathe TS, Gubert J, Krishnan T (2018). Ocular infections caused by *Scedosporium apiospermum*: a case series. Indian J Ophthalmol.

[25] Rathi HS, Venugopal A, Rengappa R, Ravindran M (2016). Scedosporium keratitis: an experience from a tertiary eye hospital in South India. Cornea.

[26] Ghosh AK, Gupta A, Rudramurthy SM, Paul S, Hallur VK (2016). Fungal keratitis in North India: spectrum of agents, risk factors and treatment. Mycopathologia.

[27] Chander J, Singla N, Agnihotri N, Arya S, Deep A (2008). Keratomycosis in and around Chandigarh: a five-year study from a north Indian tertiary care hospital. Indian J Pathol Microbiol.

[28] Thomas PA, Kaliamurthy J (2013). Mycotic keratitis: epidemiology, diagnosis and management. Clin Microbiol Infect.

[29] Sengupta J, Khetan A, Saha S, Banerjee D, Gangopadhyay N (2012). Candida keratitis: emerging problem in India. Cornea.

[30] Tuft SJ, Tullo AB (2009). Fungal keratitis in the United Kingdom 2003-2005. Eye.

[31] Chen WL, Tsai YY, Lin JM, Chiang CC (2009). Unilateral Candida parapsilosis interface keratitis after laser in situ keratomileusis: case report and review of the literature. Cornea.

[32] Dimitri T A, Joelle H, Scott D. B, Pushpanjali G, Pavan-Langston D Mandell, Douglas, and Bennett’s Principles and Practice of Infectious Diseases.

[33] Augustin JB, Variamkandi S (2021). Clinical and etiological profile of mycotic keratitis from a tertiary care centre in South India. J Evolution Med Dent Sci.

[34] Panda A, Das H, Deb M, Khanal B, Kumar S (2006). Aureobasidium pullulans keratitis. Clin Exp Ophthalmol.

[35] Chowdhary A, Perfect J, de Hoog GS (2014). Black molds and melanized yeasts pathogenic to humans. Cold Spring Harb Perspect Med.

[36] Perdomo H, Cano J, Gené J, García D, Hernández M (2013). Polyphasic analysis of *Purpureocillium lilacinum* isolates from different origins and proposal of the new species *Purpureocillium lavendulum*. Mycologia.

[37] Yuan X, Wilhelmus KR, Matoba AY, Alexandrakis G, Miller D (2009). Pathogenesis and outcome of Paecilomyces keratitis. Am J Ophthalmol.

[38] Mikami Y, Fukushima K, Arai T, Abe F, Shibuya H (1984). Leucinostatins, peptide mycotoxins produced by *Paecilomyces lilacinus* and their possible roles in fungal infection. Zentralbl Bakteriol Mikrobiol Hyg A.

[39] Leal SM, Pearlman E (2012). The role of cytokines and pathogen recognition molecules in fungal keratitis - Insights from human disease and animal models. Cytokine.

[40] Chen Y-T, Yeh L-K, Ma DHK, Lin H-C, Sun C-C (2020). Paecilomyces/Purpureocillium keratitis: a consecutive study with a case series and literature review. Med Mycol.

[41] Hirst LW, Choong K, Playford EG (2014). Nontraumatic paecilomyces anterior segment infection: a pathognomonic clinical appearance. Cornea.

[42] Ali TK, Amescua G, Miller D, Suh LH, Delmonte DW (2017). Contact-lens-associated Purpureocillium Keratitis: risk factors, microbiologic characteristics, clinical course, and outcomes. Semin Ophthalmol.

[43] Todokoro D, Yamada N, Fukuchi M, Kishi S (2014). Topical voriconazole therapy of *Purpureocillium lilacinum* keratitis that occurred in disposable soft contact lens wearers. Int Ophthalmol.

[44] Arnoldner MA, Kheirkhah A, Jakobiec FA, Durand ML, Hamrah P (2014). Successful treatment of *Paecilomyces lilacinus* keratitis with oral posaconazole. Cornea.

[45] Ford JG, Agee S, Greenhaw ST (2008). Successful medical treatment of a case of *Paecilomyces lilacinus* keratitis. Cornea.

[46] Wu P-C, Lai C-H, Tan H-Y, Ma DHK, Hsiao C-H (2010). The successful medical treatment of a case of *Paecilomyces lilacinus* keratitis. Cornea.

[47] Butt GF, Recchioni A, Moussa G, Hodson J, Wallace GR (2021). The impact of the COVID-19 pandemic on microbial keratitis presentation patterns. PLoS One.

[48] Tawde Y, Singh S, Das S, Rudramurthy SM, Kaur H (2022). Clinical and mycological profile of fungal keratitis from North and North-East India. Indian J Ophthalmol.

[49] Thomas M, Varghese A, Rajan R, Ka S, Kurian P (2011). Checklist of microfungi in Kerala, India. Asian Jr of Microbiol Biotech Env Sc.

